# Positron Emission Tomography Radiotracers for Identification of Site of Recurrence in Prostate Cancer After Primary Treatment Failure

**DOI:** 10.3390/cancers17101723

**Published:** 2025-05-21

**Authors:** Ryan Bitar, Pablo Zurita, Lucia Martiniova, Amado J. Zurita, Gregory C. Ravizzini

**Affiliations:** 1 Department of Radiology and Biomedical Imaging, Yale School of Medicine, New Haven, CT 06510, USA; ryan.bitar@yale.edu; 2 Department of Biomedical Engineering, Texas A&M University, College Station, TX 77843, USA; pazulo04@gmail.com; 3 Department of Imaging Physics, The University of Texas MD Anderson Cancer Center, Houston, TX 77030, USA; lmartini@mdanderson.org; 4 Genitourinary Medical Oncology, The University of Texas MD Anderson Cancer Center, Houston, TX 77030, USA; azurita@mdanderson.org; 5 Department of Nuclear Medicine, The University of Texas MD Anderson Cancer Center, Houston, TX 77030, USA

**Keywords:** prostate cancer, PSMA, biochemical recurrence, PET, radiotracer, PSMA-radioligands

## Abstract

Despite substantial improvement in the definitive management of primary prostate cancer, a significant number of patients experience biochemical recurrence—a clinical state in which serum prostate-specific antigen (PSA) levels rise prior to the development of physical signs or symptoms. The early detection and localization of biochemical recurrence may confer eligibility for salvage therapy; therefore, imaging techniques that provide accurate disease visualization are imperative. In this review, we discuss various imaging methods for localizing disease in the context of biochemical recurrence in prostate cancer. Particularly, we describe available or investigational positron emission tomography (PET) radiotracers, such as ^18^F-FDG, ^18^F-NaF, choline (both ^18^F and ^11^C), the ^18^F-labeled amino acid derivative fluciclovine, prostate-specific membrane antigen (PSMA) radioligands, and the short peptide compound bombesin. Generally, PET radiotracers such as ^18^F-FDG, ^18^F-NaF, and ^18^F/^11^C choline have fallen out of favor because of their inferior sensitivity and/or specificity in relation to more recently developed radiotracers. ^18^F-fluciclovine has addressed these shortcomings by exploiting the upregulation of amino acid transporters in tumors; however, PSMA-targeting agents have significantly advanced the management of biochemical recurrence of prostate cancer through their high sensitivity and specificity, enabling the identification of candidates for radionuclide therapy. Investigational agents, such as bombesin-based radiotracers, may address the shortcomings of treating prostate cancer with little to no PSMA expression.

## Simple Summary

Prostate cancer is one of the most common cancers in men. Unfortunately, even when detected and treated early, many patients experience tumor recurrence. Positron emission tomography (PET) radiotracers have emerged as promising tools for detecting recurrent tumors. This review summarizes various PET imaging radiotracers, including those already in clinical use and newer compounds that could enhance the accuracy of tumor localization. This will help physicians select the most beneficial treatments for patients. By advancing imaging techniques, these tools play an important role in guiding decisions and improving patient treatment management.

## 1. Introduction

An estimated 313,780 new cases of prostate cancer are expected to be diagnosed in the United States in 2025 [1]. Owing to their relatively late age of presentation, frequent early detection, effective treatments, and typically lengthy clinical course, prostate cancer survivors often die from competing noncancerous causes [2]. The diagnosis and treatment of primary prostate cancer have vastly improved in recent decades with advances in imaging, surgical resection, radiotherapy, and hormonal and other systemic therapies. Initial diagnosis typically involves detection through elevated and increasing serum prostate-specific antigen (PSA) levels, followed by imaging and ultrasound-guided biopsy of the prostate gland [3]. Despite recent advances in diagnosis and treatment, approximately 20–40% of patients who undergo radical prostatectomy and 30–50% of patients who receive local radiation therapy experience recurrence detected by PSA within 10 years of local curative therapy [4].

Biochemical recurrence is characterized by the identification of an increase in PSA levels without signs or symptoms of local recurrence or regional or distant metastases. PSA is a highly sensitive serum tumor marker for detecting biochemical recurrence in the case of microscopic disease, as prostate cancer cells most often secrete PSA. The definition of biochemical recurrence varies based on the initial type of treatment the patient received. Shortly after radical prostatectomy, PSA should become undetectable. In this instance, detectable serum PSA is indicative of either residual prostatic tissue due to incomplete resection or the presence of locoregional or systemic metastasis [5]. Of note, a serum PSA level that remains detectable and rises rapidly is more indicative of systemic involvement rather than residual disease at the prostatectomy bed [6,7]. Alternatively, a PSA level that gradually rises after remaining undetectable for 2 years or longer most likely indicates isolated local recurrence in the prostate bed [6,7]. For patients who have undergone radical prostatectomy, the widely accepted criterion for biochemical recurrence, according to the American Urological Association, is a serum PSA level of ≥0.2 ng/mL, confirmed by a second measurement of equal or greater value [8]. Additionally, the Phoenix criteria were specifically developed for patients with prostate cancer receiving external beam radiation therapy, but they are also commonly used in patients treated with brachytherapy [9]. According to the Phoenix definition, a PSA rise of ≥2 ng/mL above the nadir PSA level after external beam radiation therapy is suspicious for biochemical recurrence [9].

Monitoring of serum PSA is the mainstay of surveillance in men who have undergone therapy for localized prostate cancer. Guidelines from the National Comprehensive Cancer Network (NCCN) recommend that serum PSA be monitored every 6–12 months for the first 5 years after therapy and annually thereafter [10]. Early detection and accurate localization of recurrent disease may play an important role in the management of this disease by promptly identifying patients eligible for salvage therapy; therefore, diagnostic imaging is a key element in the identification and localization of recurrent prostate cancer. Identifying the site of recurrent disease when the tumor burden is very low is challenging for any imaging technique [11]. Disease detection with standard imaging techniques (i.e., computed tomography [CT], magnetic resonance imaging [MRI], or bone scan) frequently fails to pinpoint the site of (especially microscopic) recurrence.

Molecular imaging via positron emission tomography (PET) aims to address this shortcoming. ^18^F-fluorodeoxyglucose (^18^F-FDG) PET is the most widely used PET radiotracer for the detection of several types of cancers; however, this radiotracer has not proven sensitive enough for the diagnosis of primary or recurrent prostate cancer. Frequently, glycolysis and thus ^18^F-FDG avidity is low in prostate cancer, unlike in many other malignancies [12]. In addition, laboratory studies have revealed that this low avidity is due to the very weak expression of glucose transporter mRNA and protein in human prostate cancer tissue [13]. An in vitro study suggested that glucose is not essential for the metabolism of androgen-dependent prostate cancer cells, as LNCaP cells in a medium containing only 0.05 g/L glucose grew at the same rate as those in a medium with 2 g/L glucose [14]. As a result, innovations in specific target-sensitive molecular imaging techniques have sought to optimize the diagnosis of recurrent disease.

In this article, we present a review of the repertoire of radiotracers currently on the market or being investigated for PET imaging of biochemically recurrent prostate cancer. PET images collected at MD Anderson Cancer Center are included.

## 2. PET Radiotracers

### 2.1. ^18^F-FDG PET

^18^F-FDG PET has historically been one of modern medicine’s most effective diagnostic tools in cancer imaging [12,15,16]. However, there is considerable heterogeneity in clinical experience with ^18^F-FDG PET in biochemically recurrent prostate cancer. In a study by Chang et al. [17], 24 patients with rising PSA levels (>4 ng/mL) after the treatment of localized prostate cancer underwent ^18^F-FDG PET, pelvic CT, and whole-body bone scans before pelvic lymph node dissection. Despite the pelvic CT and whole-body bone scan results being negative in all patients, histopathologic examination confirmed lymph node metastases in 67% of patients. At the sites of histopathologically proven metastases, 75% of the participants showed increased ^18^F-FDG uptake. Consequently, the authors suggested that ^18^F-FDG PET may provide diagnostic value in the staging of pelvic lymph nodes in the setting of negative bone scans and non-diagnostic pelvic CT findings [17]. In contrast, Yu et al. [18] revealed that ^18^F-FDG PET exhibited the lowest detection rate when compared to other conventional imaging techniques. The clinical experience presented by Schöder et al. showed a true-positive detection rate of 31% for ^18^F-FDG PET/CT in men with biochemical recurrence following prostatectomy [19], particularly among patients with higher mean PSA levels (9.5 ± 2.2 ng/mL in PET-positive vs. 2.1 ± 3.3 ng/mL in PET-negative cases), indicating that ^18^F-FDG may not be sensitive enough for patients with lower PSA values [19]. Liu et al. evaluated prostate cancer localization using ^18^F-FDG PET in 24 treatment-naïve patients and reported only 4% sensitivity for detecting organ-confined prostate cancers [15]. Altogether, the data indicate that ^18^F-FDG PET/CT has limitations in detecting early recurrent prostate cancer, likely due to low uptake by prostate cancer cells [15,16,19,20]. ^18^F-FDG PET/CT may also be limited by false positives due to inflammatory conditions like prostatitis [21]. However, ^18^F-FDG may prove distinctly useful for detecting particularly aggressive forms of recurrent prostate cancer [22], often characterized by a high Gleason score and variant pathological features associated with lineage plasticity in the cancer cells [23].

### 2.2. ^18^F-NaF PET

^18^F sodium fluoride (^18^F-NaF) is a bone-seeking PET agent that accumulates in skeletal lesions with increased blood flow and osteoblastic activity via fluoride ion exchange within the bone matrix, with a preference for sites of newly mineralizing bone. ^18^F- is exchanged for OH-, transforming the bone matrix typically constituted by hydroxy-apatite into one modified with fluoro-apatite. Osteoblastic primary malignancies and skeletal metastases often show high uptake of ^18^F-NaF [24]. ^18^F-NaF PET/CT imaging demonstrates higher sensitivity and specificity in the evaluation of patients with suspected bone metastases than ^99m^Tc-disphosponate bone scintigraphy; however, the mechanism of radiotracer localization is nonspecific, and increased ^18^F-NaF uptake can be seen in benign osteoblastic processes, posing challenges to imaging interpretation and potential false positives [25]. ^18^F-NaF is approved by the United States Food and Drug Administration (FDA) as a bone imaging agent to define sites of altered osteogenic activity and was used clinically at some centers until December 2017, in part due to a decision by the Centers for Medicare and Medicaid Services (CMS) to reimburse ^18^F-NaF PET/CT imaging through the National Oncologic PET Registry (NOPR) [26]. In 2018, the CMS determined that the evidence to support reimbursement for ^18^F-NaF PET/CT imaging was insufficient, and the utilization of the radiotracer has decreased significantly.

A study conducted by Yoon et al. [27] examined the association between PSA levels and PSA elevation kinetics to predict ^18^F-NaF PET/CT positivity for first bone metastatic recurrence. Suspicious osseous metastases were identified in 22.2% of patients with biochemical recurrence after prostatectomy, in association with higher mean PSA values, shorter PSA doubling times (PSADT), and higher PSA velocity (PSAV). PSAV was the only parameter showing statistical significance for predicting positive ^18^F-NaF PET/CT images [27]. Jadvar et al. [28] evaluated 37 patients with biochemical recurrence of prostate cancer with ^18^F-NaF PET/CT and ^18^F-FDG PET/CT. The true-positive detection rate of ^18^F-NaF PET/CT for osseous metastases was 16.2%. ^18^F-FDG PET/CT detected metastases in only 8.1% of patients (one of whom only demonstrated metastatic lymph node involvement rather than osseous metastasis, which would not be detected on ^18^F-NaF PET/CT). Poulsen et al. [29] compared four imaging modalities—^18^F-NaF PET/CT, MRI, Technetium 99m-methyl diphosphonate (^99m^Tc-MDP) whole-body (WB) bone scan, and ^18^F-fluoromethylcholine—in 50 patients with biopsy-proven prostate cancer. The specificity of ^99m^Tc-MDP WB, and ^18^F-fluoromethylcholine were 82% and 91%, respectively, when compared to the MRI as a reference. However, the main finding of this study was the low specificity of ^18^F-NaF PET/CT (54%) for bone metastasis as compared with that of MRI. In contrast, the sensitivity of ^18^F-NaF PET/CT was remarkably high, at 93%. The investigators concluded that ^18^F-NaF PET/CT is inadequate as a stand-alone examination for the effective detection of bone metastasis due to high numbers of false positives attributable to benign degenerative or inflammatory lesions [29]. Madsen et al. [30] found that the most frequent locations of equivocal foci of ^18^F-NaF PET/CT uptake were the pelvis and the ribs, with only 25% of these lesions representing true bone metastasis. Agrawal et al. [31] assessed 120 patients with suspected prostate cancer recurrence or disease progression based on serum PSA or clinical evaluation and found the overall sensitivity, specificity, and accuracy of ^18^F-NaF PET/CT in the detection of metastatic skeletal lesions to be 96.84, 69.47, and 83.16%, respectively. ^18^F-NaF PET/CT provides increased sensitivity compared to planar bone scans, though its specificity is limited by possible false-positive findings from benign bone conditions [21].

**Figure 1:**
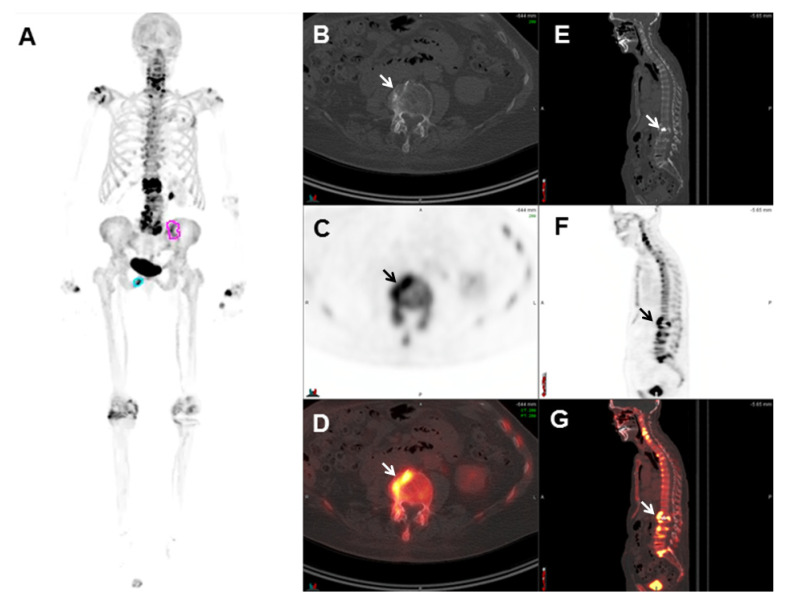
An 81-year-old patient with metastatic prostate cancer. Maximum intensity projection (MIP) images (**A**) demonstrate areas of increased ^18^F-NaF uptake in the left iliac bone (pink circle) and right inferior pubic ramus (light-blue circle), consistent with bone metastases. All other focal areas of increased ^18^F-NaF uptake in the skeleton were benign in nature. Axial CT (**B**), PET (**C**), and fused PET/CT (**D**) images at the level of the lumbar spine demonstrate focal increased ^18^F-NaF localizing to degenerative changes (arrows). Sagittal CT (**E**), PET (**F**), and fused PET/CT (**G**) images demonstrate focal increased ^18^F-NaF localizing to degenerative changes in the spine. The patient also has a fragility fracture involving L1 status post vertebroplasty, with intense surrounding activity (arrows). Frequently encountered degenerative changes pose a challenge in the interpretation of ^18^F-NaF PET/CT images.

Figure 1 presents an example of a patient with prostate cancer with increased ^18^F-NaF uptake in bone metastases. Therefore, given two major limitations of ^18^F-NaF PET/CT—(1) its limited specificity in identifying benign versus malignant osteoblastic activity and (2) its inability to evaluate for soft tissue and visceral lesions—other PET radiotracers, such as choline, fluciclovine, and PSMA radioligands have gained popularity over ^18^F-NaF.

### 2.3. ^18^F/^11^C-Choline PET

The limitations of ^18^F-FDG PET for prostate cancer detection stimulated the development of alternative radiotracers, such as choline PET. Choline is a key component of phospholipids in cell membranes. Particularly, choline undergoes phosphorylation by choline kinases to form phosphorylcholine, which is typically overexpressed in prostate cancer in order to support its malignant transformation [32]. Tissue uptake of ^11^C-choline is rapid after intravenous injection, as is its clearance from the blood [33]. After the absorption of radioactivity into a tissue, the rate of tissue uptake remains constant, with a decay correction occurring 5–40 min after injection in most organs [34].

**Figure 2:**
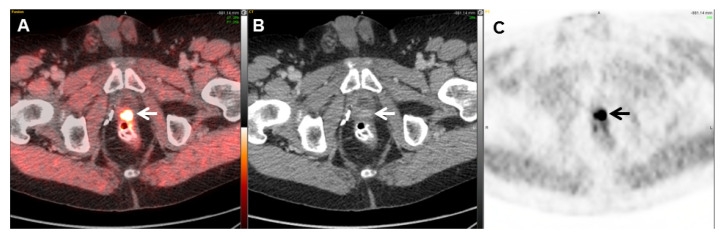
A 71-year-old man with prostate adenocarcinoma status post radical prostatectomy who had biochemical recurrence (PSA = 2.2 ng/mL). Axial fused PET/CT (**A**), CT (**B**), and PET (**C**) images demonstrate focal increased ^11^C-choline at the prostate bed (maximum standardized uptake value [SUVmax] = 5.0), compatible with tumor recurrence (arrows). The low urinary excretion of ^11^C-choline facilitates the evaluation of the prostatectomy bed.

Choline PET/CT has been a widely used technique in Europe for many years [35]. Choline can be radiolabeled with either Fluorine-18 or Carbon-11. ^11^C-choline was approved in 2012 by the FDA for patients with biochemically recurrent prostate cancer and non-informative bone scan, CT, or MRI results [36]. Choline may optimally detect local or distant recurrence with PSA values greater than 2.0 ng/mL, as reported by Picchio et al. [37]. For PSA values of <1.0 ng/mL, a positive finding is variable depending upon factors such as initial staging and Gleason score, yielding a positive detection rate ranging anywhere from ~10–40% [38]. False-positive findings were reported in ^11^C-choline PET/CT in patients with lower serum levels of PSA. In a study of 60 patients, 11 had true-positive results and 11 had false-positive findings. Nearly all the true-positive cases had a PSA of ≥1 ng/mL, while 5 of 11 false-positive cases had a PSA level below 1 ng/mL [39]. The choline detection rate after biochemical recurrence also appears to correlate with shorter PSA doubling time and increased PSA velocity [35]. There are several trials evaluating choline PET available in the literature, and among published studies, the data are inconsistent [40]. However, one meta-analysis pooled an overall sensitivity of 81.8% and a specificity value of 91.4% for all sites of recurrence, yet sensitivity and specificity values varied drastically based on the site of recurrence (local, lymph nodal, or bone) [40]. A specific finding of this report was that the sensitivity for local recurrence was relatively low; subsequently, its authors considered multiparametric MRI to be superior to choline PET in the diagnosis of local disease [40]. Due to ^11^C’s relatively short half-life (20.3 min) and the requirement of an on-site cyclotron, the implementation of ^11^C choline has posed challenges for some institutions. On the contrary, ^18^F has a half-life of 109.7 min, allowing for much easier distribution [25]. ^11^C-choline also demonstrates a significantly faster uptake time of 5 min upon administration, whereas ^18^F-choline typically displays a widely variable uptake time averaging 24 min [25]. Regarding local post-treatment recurrence, while ^18^F-choline exhibits significant sensitivity and specificity, the variable tendencies of ^18^F-choline in urinary accumulation may compromise the proper evaluation of the prostatectomy bed [38]. Therefore, ^11^C-choline may carry the advantage of detecting local recurrence, as it is mainly eliminated through the intestinal tract, with less than 2% of the injected dose excreted in the urine [38] (Figure 2). However, in the case of metastatic recurrence, a meta-analysis by von Eyben and Kairemo et al. determined that neither ^18^F-choline nor ^11^C-choline demonstrated a significant superiority in detection rate. Thus, both radiotracers seem to exhibit similar efficacy for the detection of metastatic lesions [25].

In a study by Deconinck et al. [41] evaluating the diagnostic accuracy of ^11^C-choline PET/CT in the detection of lymph node metastases, 30 patients who underwent radical prostatectomy and showed some level of positive nodal involvement on ^11^C-choline PET/CT were subjected to lymph node dissection. The reported sensitivity, specificity, positive predictive value (PPV), and negative predictive value (NPV) were 23%, 95%, 55%, and 82%, respectively, suggesting limited capabilities in localizing the degree of nodal involvement. However, Guo et al. [42] evaluated the detectability of bone metastases in a meta-analysis and concluded that choline had higher sensitivity and specificity (89% and 98%) and better performance than conventional ^99m^Tc-phosphonate bone scanning (which had a sensitivity and specificity of 71% and 91%, respectively). Similar results were found by de Leiris et al. [43], who investigated whether using ^99m^Tc-MDP for the detection of bone metastases in 115 patients with biochemical recurrence would provide more diagnostic information than ^18^F-choline PET/CT alone. The study confirmed that ^99m^Tc-MDP does not provide additive diagnostic information over concomitant ^18^F-choline PET/CT. The sensitivity and specificity of ^99m^Tc-MDP WB were 86.7% and 98.8%, compared to 93.3% and 100% for ^18^F-choline PET/CT [43].

**Figure 3:**
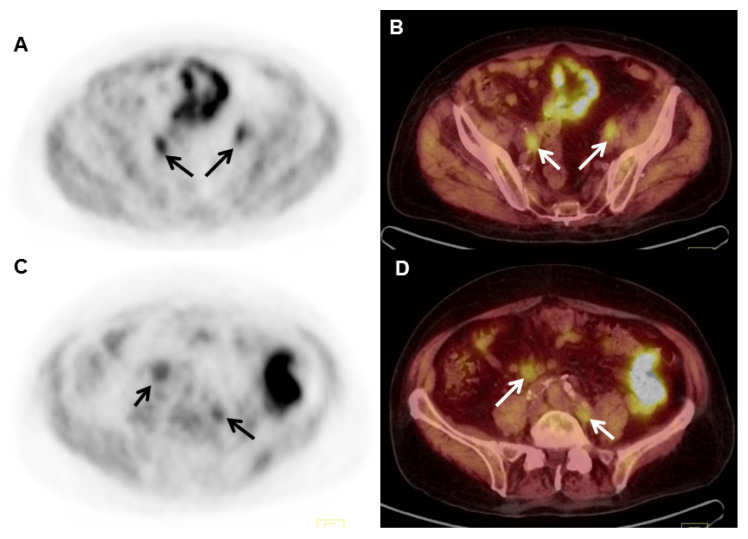
An 83-year-old man with prostate adenocarcinoma status after radical prostatectomy and biochemical recurrence (PSA = 6.3 ng/mL). Axial PET/CT (**A**) and axial fused PET/CT (**B**) images demonstrate increased ^18^F-choline activity (SUVmax = 5.4) localizing to enlarged lymph nodes in the bilateral external iliac regions, consistent with nodal metastases (arrows). Axial PET (**C**) and axial fused PET/CT (**D**) images demonstrate focal increased ^18^F-choline activity (SUVmax = 4.5) localizing to enlarged retroperitoneal lymph nodes, compatible with nodal metastases (arrows).

While ^11^C-choline and ^18^F-choline (Figure 3) both represent valuable methods for the detection of recurrence sites, the localization of the extent of some cases of biochemical recurrence remains difficult to detect, particularly at low PSA levels. This may be attributable to the relatively small tumor burden and slow membrane metabolism of some prostate cancer cells [44]. Nonetheless, studies on choline PET have stimulated further development of alternative radiotracers.

### 2.4. ^18^F-Fluciclovine (FACBC) PET

^18^F-fluciclovine (Axumin^®^; Blue Earth Diagnostics, Burlington, MA, USA), chemically abbreviated as 3-^18^FACBC, was approved by the FDA in May 2016 as a PET radiotracer for patients with suspected recurrence of prostate cancer based on elevated PSA levels following treatment [45]. Since 2018, the NCCN guidelines have included ^18^F-fluciclovine PET/CT or PET/MRI as tools in the clinical work-up of patients with recurrent prostate cancer [10].

The benefits of ^18^F-fluciclovine are similar to those of ^11^C-choline, including its rapid uptake by tumor cells as well as its minimal urinary excretion, which facilitates the evaluation of the prostatectomy bed before significant urinary accumulation is observed in the urinary bladder. In this regard, Turkbey et al. [46] showed a rapid uptake of ^18^F-fluciclovine in prostate tumors, peaking at a mean of 3.6 min after injection (range, 1.0–8.5 min) and reaching a relative plateau at 15–20 min.

^18^F-fluciclovine is a radiolabeled leucine analog. Cells take up this analog through L-type cationic amino acid transporter 1. For prostate cancer, high expression of this transporter is associated with a high Gleason score [47,48]. As seen with other discussed PET radiotracers, positive detection with ^18^F-fluciclovine is directly correlated with higher PSA levels, shorter PSA doubling time, and higher Gleason scores [49].

**Figure 4:**
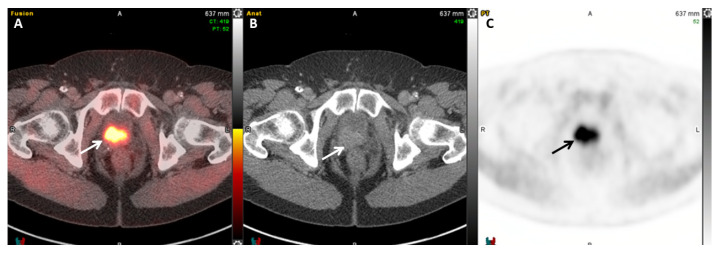
An 84-year-old man with biochemically recurrent prostate cancer 4 years following radical prostatectomy. PSA was 5.2 ng/mL at the time of PET/CT imaging. Fused axial PET/CT (**A**), axial CT (**B**), and PET (**C**), ^18^F-fluciclovine images demonstrate an area of increased radiotracer uptake (SUVmax = 13.7) corresponding to a 3.6 × 2.6 cm enhancing lesion at the prostatectomy bed invading the bladder neck and posterior wall of the urinary bladder (arrows).

Initial data from a European study indicated that ^18^F-fluciclovine may have better overall detection rates than ^11^C-choline [50] (Figure 4). However, concerns have been raised regarding the results of this trial, as a much lower dose of ^11^C-choline (3.4 MBq/kg) was employed in comparison to the dose frequently administered in the U.S. (720 MBq). A study by Schuster et al. [51] showed that, following brachytherapy or cryotherapy, a high percentage of patients were left with residual viable prostate tissue and were thus more susceptible to confounding by inflammation or hypertrophy, resulting in 90.2% sensitivity but a low specificity of 40%. Virarkar et al. [52] compared ^18^F-fluciclovine PET/CT with MRI and found that MRI had higher concordance (96%), specificity (91%), sensitivity (100%), PPV (93%), and NPV (100%) in detecting locally recurrent prostate cancer. These results indicate that ^18^F-fluciclovine PET/CT has limited added value in detecting local recurrence when compared to MRI. When an SUVmax cut-off value > 2.85 was implemented, ^18^F-fluciclovine PET/CT showed enhanced specificity and accuracy. It is also possible to use both MRI and PET/CT in conjunction [52].

A prospective multicenter trial, LOCATE, assessing the impact of ^18^F-fluciclovine PET/CT imaging in patients with biochemical recurrence of prostate cancer (median PSA 1.0 ng/mL) after primary therapy with curative intent, demonstrated ^18^F-fluciclovine avid lesions in 122 of the 213 patients (57%) [53]. This detection rate is higher than the published detection rate for ^11^C-choline. Most importantly, 126 of the 213 patients (59%) had a change in management after the scan. Furthermore, ^18^F-fluciclovine PET/CT may have a significant impact on therapy decisions during salvage radiotherapy (SRT) planning [54]. In the EMPIRE-1 trial (Emory Molecular Prostate Imaging for Radiotherapy Enhancement 1), the incorporation of ^18^F-fluciclovine PET/CT to guide final radiotherapy treatment decisions and target volume design resulted in improved failure-free survival (FFS) [55]. Similarly, a subsequent study by Lawal et al. showed that SRT planning using ^18^F-fluciclovine PET/CT was associated with significantly longer FFS than using conventional imaging alone in patients with serum PSA levels below 2 ng/mL [56]. Imaging with ^18^F-fluciclovine presents certain limitations. False-positive findings can occur in the presence of benign prostatic hyperplasia, prostatitis, and post-radiation inflammation [57]. Savir-Baruch et al. highlighted the challenges of using ^18^F-fluciclovine to evaluate local recurrence, noting a higher rate of false positives within the prostate compared to extra-prostatic regions [58]. Additionally, increased ^18^F-fluciclovine activity may be seen in non-prostatic malignancies and various benign conditions [58].

### 2.5. PSMA Radioligands

More recently, various prostate-specific membrane antigen (PSMA) radioligands were introduced clinically for PET/CT imaging, some labeled with Gallium-68 and others with Fluorine-18 [22]. Also known as glutamate carboxypeptidase II, PSMA is a 100-kDa type 2 transmembrane zinc metalloenzyme expressed in prostate epithelial cells [59]. The expression of PSMA is relatively low in normal prostate tissue; however, PSMA expression rises in prostate cancer cells. Expression also increases with higher tumor grade and is extensively upregulated in castration-resistant prostate cancer (cancer that grows even when deprived of androgen stimulation) [60].

The earliest PSMA-targeting molecule put into practice was ProstaScint (Aytu, BioScience Inc, Englewood, CO), a ^111^In-labeled version of the monoclonal antibody 7E11 [22]. ProstaScint is a conjugated murine IgG1 monoclonal antibody (7E11-C5.3) that identifies the PSMA epitope localized in the intracellular domain of PSMA. The major downside of ProstaScint imaging was that it was suboptimal for the detection of bone metastases as the antibody targets the intracellular domain of PSMA and binds only to dead or dying cells, failing to recognize viable cancer cells. ProstaScint imaging was significantly less sensitive than bone scans [61,62], which led to limited clinical adoption before its eventual discontinuation in 2018 [22].

#### 2.5.1. ^68^Ga-PSMA-11

**Figure 5:**
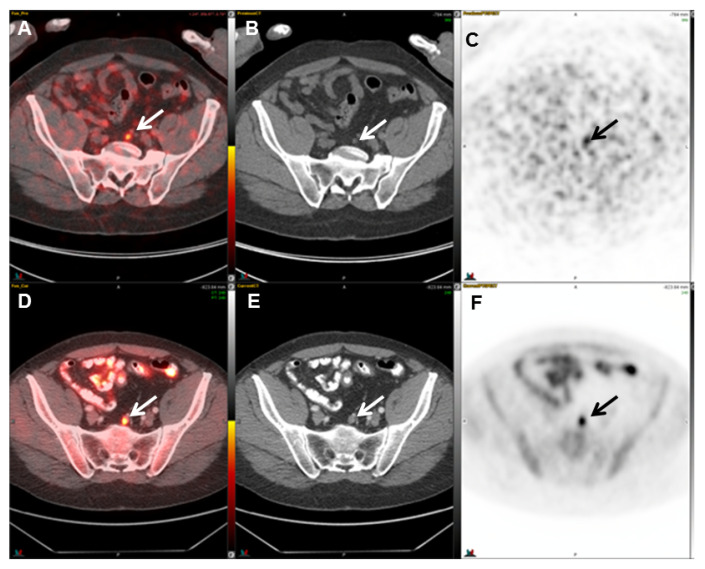
A 74-year-old man with prostate adenocarcinoma status post radical prostatectomy and biochemical recurrence. Axial fused PET/CT (**A**), CT (**B**), and PET (**C**) images demonstrate focal increased ^68^Ga-PSMA-11 activity (SUVmax = 4.2) localizing to a 0.7 cm lymph node in the pelvis (arrows), consistent with nodal metastasis. Axial fused PET/CT (**D**), CT (**E**), and PET (**F**) images of an ^11^C-choline study obtained 4 months later demonstrate focal increased ^11^C-choline activity (SUVmax = 4.8) localizing to the same lymph node, which has enlarged, measuring 1.0 cm (arrows).

Several low-molecular-weight PSMA radioligands are currently available for clinical use in the United States, and many others are undergoing testing under investigational new drug (IND) applications. The first PSMA radioligand the FDA approved in December 2020 was ^68^Ga-PSMA-11 [Glu-Urea-Lys-(Ahx)-HBED-CC]. ^68^Ga-PSMA-11 binds with high affinity to PSMA [63]. Gallium-68 (^68^Ga, half-life = 67.6 min, Eβ + max = 1.92 MeV, mean 0.89 MeV) is most frequently obtained from a Germanium-68/Gallium-68 (^68^Ge/^68^Ga) generator but can also be produced via a cyclotron [64]. Other ^68^Ga-based PSMA radioligands currently under investigation (^68^Ga-PSMA-617, ^68^Ga-PSMA I&T, and ^68^Ga-PSMA-R2, among others) demonstrate similar biodistribution and imaging characteristics amongst each other [65,66]. It is believed that PSMA, once bound by its radioligand, becomes internalized through endocytosis [67]. ^68^Ga-PSMA-11 has demonstrated high detection rates for identifying sites of biochemical recurrence in prostate cancer (Figure 5). In one study, ^68^Ga-PSMA-11 detected 88 prostate cancer lesions, including local recurrence, lymph node metastases, and lung, bone, and soft-tissue metastases, with an overall positive lesion detection rate of 71% [68]. Generally, dynamic PET signal acquisition reveals increased radiotracer uptake during late acquisition times; thus, a 60 min uptake time is recommended before imaging [63]. Increased sensitivity has been reported with delayed imaging up to 3 to 4 h after injection [69]. The overall detection rate of recurrent lesions in patients with relatively low PSA levels is significantly higher than in patients evaluated using choline PET/CT [63]. However, while ^68^Ga-PSMA-11 generally outperforms choline in the detection of local recurrence, high urinary bladder activity still presents a challenge in the detection of tumors in the prostate bed [70]. Therefore, some investigators have advocated for the use of a normal saline infusion followed by diuretics as part of the imaging protocol. Even though ^68^Ga-PSMA-11 demonstrates superiority to the more traditional ^99m^Tc-diphosphonates for the detection of bone metastasis, one evident limitation in the specificity of ^68^Ga-PSMA-11 is the presence of physiological, subtle uptake in various skeletal regions, which may result in false-positive findings [70]. ^68^Ga-PSMA-11 uptake is also observed in most organs in the abdominal region, such as the liver, colon, kidney, spleen, small intestine, and adrenal glands, which may also hinder image interpretation [70]. Since PSMA is also expressed in the neovasculature of many solid tumors, radiotracer uptake may be seen in a variety of other non-prostate malignancies [45]. Low PSMA uptake may also occur in the parasympathetic ganglia mimicking nodal metastases, most often in the celiac and stellate ganglia but also the pre-sacral ganglia [71]. It is important to note that approximately 5–10% of all prostate cancers do not display significant PSMA expression [72]. Currently, ^68^Ga-PSMA-11 PET/CT is performed in patients with prostate cancer with biochemical recurrence, even at low PSA levels. As PSA levels rise, the detection rate also increases. Detection rates of 40% have been described with PSA levels of ≤1 ng/mL, increasing to 80% with PSA levels between 1 and 2 ng/mL and reaching 90% with PSA levels of ≥2 ng/mL [73,74]. A recent study by Burgard et al. confirmed the trend, where retrospectively examined data from 115 patients with PSA levels greater than 0.2 ng/mL following radical prostatectomy were analyzed over 7 years [75]. Detection rates were higher (35%) for patients with PSA > 0.15 ng/mL compared to those with PSA between 0.1–0.15 (18.9%) and PSA < 0.1 ng/mL (21.1%). Patients with a doubling time of <12 months and a Gleason score of ≥7b had significantly higher detection rates [75]. Fendler et al. reported that ^68^Ga-PSMA-11 PET/CT demonstrated a false-positive rate of under 10%, with most false-positive uptake occurring in the prostate following radiotherapy. False-positive uptake was also observed in treated benign tissue or potentially indolent tumor remnants and various non-prostatic malignancies [76].

^68^Ga-PSMA-11 is now available commercially under the trade names Illuccix^®^ and Locametz^®^ [77]. Calais et al. [78] compared ^18^F-fluciclovine and ^68^Ga-PSMA-11 in 50 patients with early biochemical recurrence and found significantly lower detection rates with ^18^F-fluciclovine (13 positive patients) than with ^68^Ga-PSMA-11 (28 positive patients). ^68^Ga-PSMA-11 PET/CT sensitivity was 66%, and ^18^F-fluciclovine PET/CT sensitivity was 33% [78].

#### 2.5.2. ^64^Cu-DOTAGA-PSMA

In addition to ^68^Ga radioligands, other promising radionuclides for labeling PSMA, such as Copper-64 (^64^Cu, half-life = 12.7 h; Eβ + max = 0.65 MeV [17.9%]; Eβ − max = 0.57 MeV [39%]), are currently under investigation for PET imaging. ^64^Cu is cyclotron produced, and the longer half-life than that of ^68^Ga (12.7 h versus 68 min) makes ^64^Cu-PSMA more suitable for commercial distribution. One of the drawbacks of Cu-64 is its low positron emission yield (17.9% only), which may decrease the signal-to-noise ratio. Nonetheless, ^64^Cu for PET imaging is gaining popularity. Singh et al. [79] reported their initial experience with ^64^Cu-PSMA PET/CT in prostate cancer. Nine patients were evaluated for restaging following the elevation of PSA. ^64^Cu-PSMA was found to be safe for clinical use and demonstrated a high theranostic potential for molecular imaging in prostate cancer. With promising imaging characteristics and with a possible imaging window of up to 17 h after administration, ^64^Cu-PSMA PET/CT could potentially be used as an alternative agent to ^68^Ga-PSMA PET/CT [79].

Mirzaei et al. retrospectively compared ^64^Cu-DOTAGA-PSMA imaging to ^18^F-PSMA PET/CT imaging in patients with prostate cancer with biochemically recurrent disease [80]. In that study, the results were comparable between both imaging agents. The total detected number of lesions was higher with ^64^Cu-DOTAGA-PSMA (209 vs. 191). A total of 41/50 patients (PSA range: 0.01–0.7 µg/L) imaged with ^64^Cu-DOTAGA-PSMA and 44/50 (PSA range: 0.01–4.2) with ^18^F-PSMA had at least one PSMA positive lesion. A noteworthy finding is that the median SUV mean for ^18^F-PSMA was slightly higher in the liver (11.8 vs. 8.9) [80], suggesting that ^64^Cu-DOTAGA-PSMA could potentially detect more liver metastases. Different chelator moiety choices affect the pharmacokinetics of the radiopharmaceutical [81]. PET studies conducted in the past demonstrated the potential of ^64^Cu-NODAGA-PSMA for the detection of residual disease in patients with recurrent or primary progressive prostate cancer. The performance of ^64^Cu-DOTAGA-PSMA was better than that of ^64^Cu-NODAGA-PSMA PET/CT [82].

**Figure 6:**
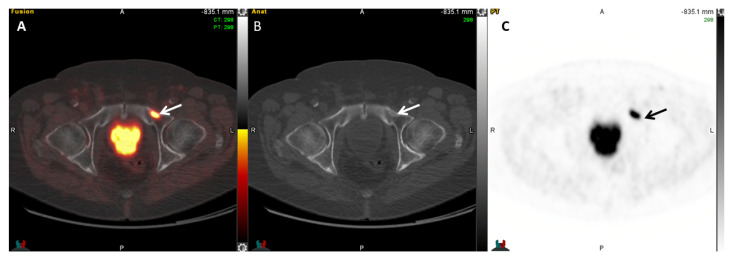
A 74-year-old man with biochemically recurrent prostate cancer with serum PSA of 2.7 ng/mL. Fused axial PET/CT (**A**), axial CT (**B**), and PET (**C**) images demonstrate a focus of increased ^64^Cu-PSMA I&T uptake in the left superior pubic ramus (SUVmax 16.2, arrows), compatible with a bone metastasis.

An additional conjugate is available for imaging and therapy through PSMA targeting (PSMA-I&T). PSMA-I&T can be radiolabeled with ^64^Cu for imaging or with ^177^Lu for therapy. Lee et al. evaluated the feasibility of ^64^Cu labeling in vivo and confirmed the specificity for PSMA targeting, with the highest uptake time at 48 h, leading them to consider ^64^Cu-PSMA-I&T in future clinical trials [83]. A poster describing the first-in-human study was presented at the 2024 Mid-Winter annual meeting of the Society of Nuclear Medicine and Molecular Imaging (SNMMI), showing that ^64^Cu-PSMA-I&T injection was safe and well tolerated and gave clinically relevant results [84]. Twelve patients were enrolled in Phase I, and 26 patients were enrolled in Phase II with histologically proven prostate adenocarcinoma and at least one extra-prostatic site of the disease suspected based on prior imaging or tissue sampling. There were no drug-related adverse events reported in any patients or significant changes in laboratory assessments, vital signs, or electrocardiograms [84]. Consequently, Curium (St. Louis, Missouri) announced the enrollment of patients into the Phase III clinical trials SOLAR-STAGE (NCT06235151) and SOLAR-RECUR (NCT06235099) evaluating the diagnostic performance of ^64^Cu-PSMA-I&T. SOLAR-RECUR examines the diagnostic performance of ^64^Cu-PSMA-I&T PET/CT in men with suspected biochemical recurrence of prostate cancer following radical prostatectomy or radiation therapy with a curative intent following radical prostatectomy [85]. The SOLAR-STAGE trial focuses on the diagnostic performance of ^64^Cu-PSMA-I&T in men recently diagnosed with unfavorable intermediate-, high-, or very-high-risk prostate cancer and those who plan on undergoing radical prostatectomy and pelvic lymph node dissection. Figure 6 represents an example of a ^64^Cu-PSMA-I&T PET/CT image.

#### 2.5.3. ^64^Cu-SAR-bisPSMA

A new bivalent PSMA inhibitor SAR-bisPSMA was introduced by the Donnelly group and was subsequently conjugated with the ^64^Cu/^67^Cu diagnostic/therapeutic pair [86,87]. Zia et al. [86] first presented SAR-bis PSMA as a molecule consisting of a macrobicyclic sarcophogine radiochelation complex and a bivalent ligand with two PSMA-targeting groups, which demonstrates selective binding to PSMA-positive LNCaP human prostate adenocarcinoma cells. Several clinical trials using ^64^Cu-SAR-bisPSMA for prostate cancer diagnosis and ^67^Cu-Sar-bisPSMA for therapy were started in 2021. PROPELLER (NCT04839367) was a Phase I trial that investigated ^64^Cu-SAR-bisPSMA in 30 men with untreated, histopathologically proven primary prostate cancer with intermediate- to high-risk features [88]. ^64^Cu-SAR-bisPSMA was reported to be safe and effective for detecting PSMA-expressing lesions. An optimal dose of 200 MBq was determined for future studies [89]. Encouraging reports from the PROPELLER trial led to the initiation of the Phase III CLARIFY trial (NCT06056830), a multicenter diagnostic study of ^64^Cu-SAR-bisPSMA PET in patients with high-risk prostate cancer prior to radical prostatectomy [90]. Recruitment for this trial is currently ongoing. Clarity Pharmaceuticals Ltd. has additionally completed several clinical trials in patients with biochemical recurrence of prostate cancer following definitive therapy using ^64^Cu-SAR-bisPSMA (COBRA- NCT05249127) and a Phase I/IIa trial, Theranostic Study of ^64^Cu-SAR-bisPSMA and ^67^Cu-SAR-bisPSMA for Identification and Treatment of PSMA-expressing Metastatic Castrate Resistant Prostate (SECuRE-/NCT04868604). This latter study is being conducted in three phases: dosimetry, dose escalation, and dose expansion [91].

#### 2.5.4. ^18^F-DCFPyL

**Figure 7:**
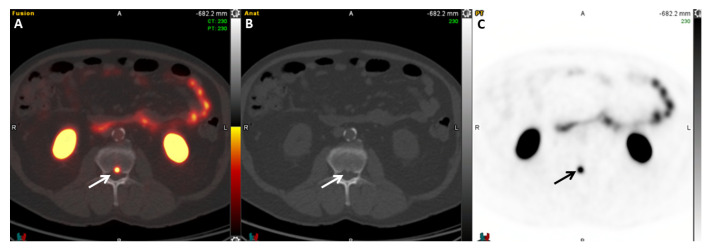
A 69-year-old man with biochemically recurrent prostate cancer with a PSA of 0.1 ng/mL. Fused axial PET/CT (**A**), axial CT (**B**), and PET (**C**), ^18^F-DCFPyL PSMA images demonstrate a focus of increased radiotracer uptake in L2 (SUVmax = 19.1), compatible with a bone metastasis (arrows).

Fluorinated radiotracers targeting PSMA have also emerged. 2-(3-(1-carboxy-5-[(6-[^18^F]fluoro-pyridine-3-carbonyl)-amino]-pentyl)-ureido)-pentanedioic acid (^18^F-DCFPyL) was proposed [92,93]. Initial patient studies revealed that the radiotracer was well tolerated, exhibiting only three grade 1 adverse events (per the Common Terminology Criteria for Adverse Events) among a total of nine patients. High physiologic activity was observed in the salivary glands, kidneys, and urinary bladder, which may also occur with other PSMA-targeted radioligands. Favorable tumor uptake, rapid plasma clearance, and low accumulation in the liver and muscle tissues were reported. While optimal visualization was noted at the 1 h timepoint, the authors recommended examining later PET images for patients with biochemical recurrence; however, this recommendation brought up a concern, as imaging past 1 h may demonstrate increased radiotracer activity in the urinary bladder, obscuring any local lesions [93]. In a study that directly compared ^18^F-DCFPyL to ^68^Ga-PSMA-11, the fluorinated compound proved to be a promising alternative. All lesions detected by the ^68^Ga compound were detected by ^18^F-DCFPyL, with a significantly higher SUVmax and generally better tumor-to-background ratios, except when the reference organ was the liver or mediastinum (Figure 7). Furthermore, additional suspicious lesions plausible for metastases were detected in 3/14 patients using this compound versus ^68^Ga-PSMA-11 [94]. Based on the OSPREY [95] and CONDOR [96] studies, ^18^F-DCFPyL (Piflufolastat F-18 (PYLARIFY^®^)) was approved by the FDA in May 2021 for PET detection of PSMA-positive lesions in men with prostate cancer with suspected metastasis [97]. Subsequently, the 2022 NCCN guidelines incorporated the use of ^68^Ga-PSMA-11 and ^18^F-DCFPyL PET/CT and PET/MR for detecting recurrences, since PSMA PET ligands present better sensitivity and specificity than conventional imaging (CT or MRI) for detecting micro-metastatic prostate cancer [98].

Ulaner et al. [99] prospectively assessed 92 patients and compared ^18^F-DCFPyL PET/CT uptake with histopathologic proof for targeting PSMA in lesions suspected of being distant metastases. Among these, 62% demonstrated DCFPyL-avid lesions suspicious for recurrence. Biopsies were performed in 65% of patients, with 89% of the samples confirming malignancy; 11% were benign, especially in solitary foci in the ribs and pelvic bones and along the pelvic nodal chains. From these, 50% of the biopsied rib foci were determined to be false-positive findings, and 29% of biopsied pelvic osseous foci were false positives [99].

#### 2.5.5. ^18^F-PSMA-1007

**Figure 8:**
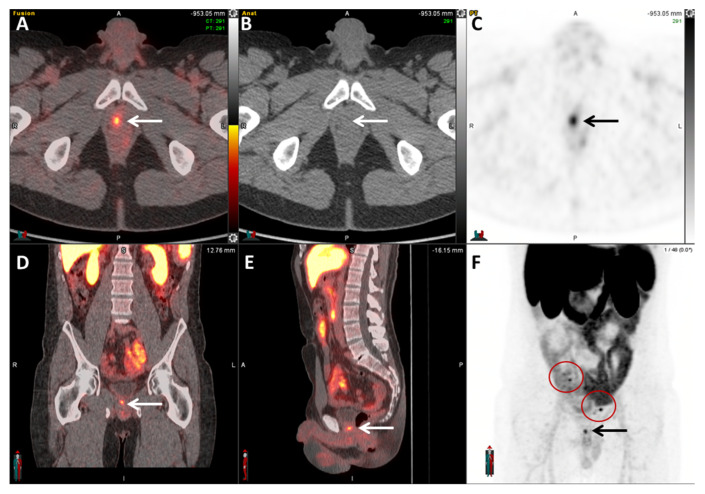
A 53-year-old man with biochemically recurrent prostate cancer with PSA of 0.4 ng/mL. Axial fused PET/CT (**A**), axial CT (**B**), axial PET (**C**), coronal PET (**D**), sagittal PET (**E**), and MIP images (**F**) demonstrate a focal area of increased ^18^F-PSMA-1007 uptake at the right aspect of the bladder neck (arrows, SUVmax = 7.3). The low urinary excretion of ^18^F-PSMA-1007 facilitates the evaluation of the prostatectomy bed. MIP images demonstrate additional foci of increased uptake in the pelvis bilaterally (circles), compatible with nodal metastases.

More recently, another promising ^18^F-labeled PSMA radioligand has been investigated in humans, ^18^F-PSMA-1007 [100]. Prostate tumors and normal PSMA-expressing tissues (the parotid glands, liver, spleen, and kidneys) showed similar biodistribution patterns for ^18^F-PSMA-1007 and ^68^Ga-PSMA-11 [101], but ^18^F-PSMA-1007 demonstrated a statistically significant increase in SUV uptake in intraprostatic lesions relative to the uptake by the urinary bladder. Furthermore, due to its slightly higher lipophilic nature, ^18^F-PSMA-1007 (Figure 8) is primarily excreted via the hepatobiliary route, causing a greater accumulation in the gallbladder and intestines when compared with ^68^Ga-PSMA-11 [101], and reduced urinary clearance (1.2% injected dose over 2 h), enabling superior assessment of tumor recurrence in the prostatectomy bed. In contrast, the urinary excretion of ^18^F-DCFPyL, ^68^Ga-PSMA-11, and ^68^Ga-PSMA-617 is remarkably high (>10% injected dose over 2 h). According to a study by Pattison et al. [102], ^18^F-PSMA-1007 detected additional bladder wall invasive and lymph node metastatic lesions near the ureter due to the lack of urinary tract tracer retention. In turn, ^18^F-PSMA-1007 was reported to show a significantly higher SUVmax for benign bone lesions than ^68^Ga-PSMA-11. Rauscher et al. found that in patients with biochemical recurrence, the SUVmax of ^18^F-PSMA-1007 in benign lesions was greater than that of ^68^Ga-PSMA-11 (medium SUVmax = 5.3 vs. 4.4) [103]. Congruent with this finding, the rate of false-positive bone uptake for ^18^F-PSMA-1007 was higher [104].

Seifert et al. [105] investigated the nonspecific bone uptake with ^18^F-PSMA-1007 in 383 patients who underwent both ^18^F-PSMA-1007 and ^68^Ga-PSMA-11 in an intraindividual comparison. They reported that in tumors with an SUV > 4 and PSA levels > 5 ng/mL during biochemical recurrence, most focal bone uptake identified as false-positive finding was located in the ribs and pelvis and attributed to nonspecific bone uptake. A total of 33 nonspecific bone uptake regions were identified as false-positive on ^18^F-PSMA-1007, while ^68^Ga-PSMA-11 showed four occurrences of false-positive bone uptake [105]. In such cases where the clinical likelihood of metastatic disease is low, focal bone uptake observed on ^18^F-PSMA-1007 should be interpreted with caution. Careful consideration should be given to both anatomical localization and the overall clinical context. Nonetheless, the current incidence of bone metastases detected with ^18^F-PSMA-1007 in routine clinical practice was not higher, suggesting that experienced nuclear medicine physicians are generally able to recognize and appropriately interpret unspecific bone uptake [105].

Due to the longer half-life of ^18^F and its superior energy characteristics, fluorinated PSMA radiotracers have proven to be an effective alternative to ^68^Ga-PSMA-based compounds for commercial distribution [94].

#### 2.5.6. ^18^F-rhPSMA-7.3

New classes of PSMA-binding compounds have been developed that can efficiently be labeled with ^18^F or radioactive metal isotopes, such as radiohybrid PSMA (rhPSMA) ligands from Blue Earth Diagnostics. For instance, ^18^F-rhPSMA-7.3 has demonstrated rapid clearance in the blood pool, liver, and kidney, as well as a high level of accumulation in tumors [106]. Clinical data indicate that ^18^F-rhPSMA-7.3 has a lower urinary excretion than ^18^F-DCFPyL and ^68^Ga-PSMA-11 [107]. In the SPOTLIGHT clinical trial (NCT04186845), ^18^F-rhPSMA-7.3 (flotufolastat) was evaluated for diagnostic performance and safety. A patient-level verification of ^18^F-flotufolastat in recurrent prostate cancer patients demonstrated detection rates of 51% to 54%, with an overall detection rate of 81% in 389 patients studied with an evaluable scan [108,109]. Relatively high false-positive findings were reported in the prostate bed, pelvic lymph nodes, and extra-pelvic sites in the SPOTLIGHT clinical trial (Supplemental Table B4) [109]. However, the PPV was limited by the predominant use of conventional imaging as the standard of truth. This limitation likely contributed to some PET-positive lesions being considered false positives that might have been identified as true positives if a more sensitive standard had been used. Retrospectively analyzed studies on the detection rate and PPV of ^18^F-rhPSMA-7 PET/CT in 532 patients [108,109,110] revealed that a total of 23 ^18^F-rhPSMA-7 PET/CT positive lesions in the histopathology-confirmed cohort were identified as non-prostatic-related tissue. A total of 13% of the findings were false positives, occurring in the lung nodes, bone, prostate bed, and visceral organs [110].

Flotufolastat F18 (POSLUMA^®^) was FDA-approved in May 2023 for PET imaging of patients with prostate cancer with suspected metastases or high serum prostate-specific antigen levels and suspected recurrence [111].

**Figure 9:**
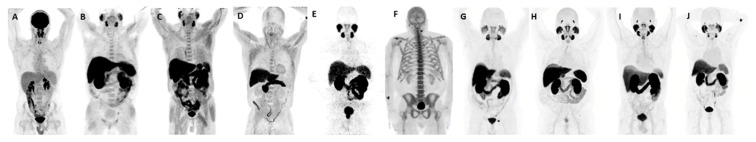
Maximum intensity projection (MIP) images obtained in different patients demonstrating the differences in normal biodistribution (or with small tumors) of ^18^F-FDG (**A**), ^11^C-choline (**B**), ^18^F-choline (**C**), ^18^F-fluciclovine (**D**), ^68^Ga-PSMA-11 (**E**), and ^18^F-NaF (**F**). Foci of increased activity in the cervical spine, thoracic spine, and right shoulder seen on the ^18^F-NaF image (**F**) are compatible with degenerative changes. The focus of increased uptake in the right forearm is the injection site. The ^64^Cu-PSMA I&T image (**G**) demonstrates a focus of increased uptake in the left superior pubic ramus (SUVmax 16.2), consistent with a bone metastasis, with an additional focus of increased uptake in the left pelvis, corresponding to a 0.8 cm nodal metastasis (SUVmax 12.5). (**H**) ^18^F-PSMA-1007, (**I**) ^18^F-DCFPyL, and (**J**) ^18^F-rhPSMA-7.3.

Overall, the overexpression of PSMA by prostate cancer cells has proved to be a useful diagnostic PET biomarker to evaluate biochemical recurrence; however, limitations in its implementation must be considered. The normal biodistribution of PSMA-targeting agents results in marked uptake in the kidneys, ureters, urinary bladder, salivary glands, and small bowel and moderate uptake in the liver and spleen (Figure 9). Uptake is also present in benign osteoblastic processes (osteoarthritis, Paget’s disease, and healing fractures) and neovascularization from other nonprostatic malignancies (renal cell carcinoma, glioblastoma, and hepatocellular carcinoma, for instance). Moreover, <10% of prostate cancer cells do not sufficiently overexpress PSMA, and therefore, new targets should be considered [71].

### 2.6. Bombesin

Bombesin has been suggested as such alternative target to PSMA. Bombesin is a 14-amino-acid peptide discovered on the skin of Bombina bombina and Bombina variegata frogs in 1970 [112]; the mammalian analog, gastrin-releasing polypeptide (GRP), was isolated later [112]. GRP receptors may be overexpressed in prostate cancer, including smaller and lower-grade disease [113].

^68^Ga-labeled DOTA-4-amino-1-carboxymethyl-piperidine-DPhe-Gln-Trp-Ala-Val-Gly-His-Sta-Leu-NH2 ^68^Ga-RM2, or ^68^Ga-DOTA-bombesin (formerly known as BAY86-7548) is a synthetic bombesin receptor antagonist that targets gastrin-releasing peptide receptors [114]. These receptors are highly overexpressed in several human tumors, including 63–100% of prostate cancers [115,116,117]. ^68^Ga-RM2 and ^68^Ga-PSMA-11 target different biological processes and may, therefore, provide different diagnostic information in patients with biochemically recurrent prostate cancer. Minamimoto et al. [118] assessed both radiotracers in patients with a rising PSA and found that both tracers presented similar localization patterns in lymph nodes, seminal vesicles, and bone marrow. Ultimately, ^68^Ga-RM2 cleared relatively slowly from the major vessel blood pool, complicating the identification of mediastinal and supraclavicular lymph nodes [118]. In 2013, an in vivo study examined the efficacy of variable bombesin analogs radiolabeled with either ^64^Cu or ^18^F. ^18^F-AlF-NODAGA-RM1 exhibited excellent stability as well as favorable biodistribution and tumor uptake in mouse xenografts [119]. One human study investigated the in vivo imaging of ^68^Ga-radiolabeled BAY86-7548 bombesin analog. This radiotracer demonstrated high accuracy (83%) in primary and locally recurrent disease; however, metastatic lesions did not demonstrate a sensitivity rate (70%) superior to that of choline tracers [120]. Additionally, this compound only entered clinical testing recently, and thus available data are still limited. GRP receptor-targeting radiopharmaceuticals may provide an important complementary radioligand therapy for cancers with no or low PSMA expression and for tumors with heterogeneous expression of target receptors [121]. Clarity Pharmaceuticals is developing one such agent, known as SAR-BBN, which may provide a potential imaging option (^64^Cu-SAR-Bombesin) and treatment option (^67^Cu-SAR-Bombesin) for patients with little or no PSMA [122]. According to preliminary clinical data, ^64^Cu-SAR-BBN was able to detect lesions in 32% (8/25) of patients with biochemical recurrence and negative/equivocal PSMA PET scans. This study was also remarkable in showing a false-positive rate of only 8% (2/25) of patients [123]. The SABRE trial (NCT05407311) is a Phase II, non-randomized, open-label study of ^64^Cu-SAR-BBN administered to patients with recurrence of prostate cancer following definitive therapy [123]. Separately, the COMBAT trial (NCT05633160) is an open-label Phase I/IIa clinical trial investigating the theranostic pair ^64^Cu-SAR-BBN and ^67^Cu-SAR-BBN for concurrent diagnosis and therapy in patients with metastatic castration-resistant prostate cancer who are ineligible for treatment with ^177^Lu-PSMA-617 [124].

### 2.7. PSMA Radioligand Therapy

PSMA radioligands can also be used as a theranostic tool, serving as a targeted systemic radiotherapy option and an imaging tool for post-therapy response. For instance, Baum et al. [125] conducted a study on 56 patients with metastatic castrate-resistant prostate cancer (mCRPC) who underwent peptide radioligand therapy with ^177^Lu-PSMA. Patients with evidence of an increased ^68^Ga-PSMA-11 uptake on the preceding PET/CT were given an infusion of ^177^Lu-PSMA at a median dose of 5.8 GBq. Subsequently, follow-up ^68^Ga-PSMA-11 PET/CT was employed to evaluate molecular changes and clinical response to therapy. Eighty percent of patients demonstrated a decrease in PSA serum levels, and a median progression-free survival rate of 13.7 months was observed. Thus, ^68^Ga-labeled radioligands could be used as a tool not only for the selection but also for monitoring response in patients receiving targeted radionuclide therapy.

PSMA-11 has been further modified to create a new ligand called PSMA-617 with an even higher binding affinity to PSMA receptors. ^177^Lu-PSMA-617 was gradually introduced in Europe as radioligand therapy for patients with metastatic prostate cancer starting in 2013 [126]. To assess the efficacy and safety of ^177^Lu-PSMA-617, Rahbar et al. performed a retrospective analysis in 145 patients at 12 centers with mCRPC [127]. Treatment consisted of one to four therapy cycles and an activity range of 2–8 GBq per cycle. ^177^Lu-PSMA-617 confirmed favorable safety and high efficacy [128]. In March 2022, the FDA approved ^177^Lu-PSMA-617 (Pluvicto^®^, lutetium L-177 vipivotide tetraxetan), for the treatment of adult patients with PSMA-positive mCRPC previously treated with androgen receptor pathway inhibition and taxane-based chemotherapy [129]. In March 2025, the FDA broadened the approval of Pluvicto^®^ for the treatment of mCRPC in adults who have experienced progression despite androgen receptor pathway inhibitor (ARPI) therapy and are deemed suitable for postponing taxane-based chemotherapy [130]. The FDA granted approval based on the results of the randomized Phase 3 controlled trial PSMAfore (NCT04689828). This trial was conducted at 74 multinational centers and found that ^177^Lu-PSMA-617 prolonged radiographic progression-free survival (rPFS) compared with a change in ARPI in patients with PSMA-positive mCRPC who had progressed on a previous ARPI (11.6 months among patients who received ^177^Lu-PSMA-617 vs. 5.6 months among patients who received a change in ARPI), resulting in a 59% reduction in the risk of radiographic progression or death [131].

Investigations on radionuclide therapy have not been limited to beta-emission. Actinium-225 (^225^Ac) is an alpha-emitting radionuclide with higher energy, shorter range, and longer half-life (9.92 days) than ^177^Lu and is currently being evaluated for the treatment of metastatic castration-resistant prostate cancer [132]. Although much needs to be learned to validate the safety and efficacy profile of ^225^Ac, Ma et al. [132] conducted a meta-analysis on 201 patients with mCRPC who failed second/third-line therapy (including ^177^Lu-PSMA-617) and underwent treatment with ^225^Ac-PSMA-617. Eighty-seven percent of study participants experienced a decrease in PSA, and overall survival and progression-free survival were 12.5 months and 9.1 months, respectively. With the development of new agents for targeted radioligand therapy, the need for widely available PSMA radioligands for imaging of prostate cancer will become even more important.

## 3. Hybrid Imaging and Future Research

### 3.1. PET/MRI PSMA Imaging

Recent developments in imaging instrumentation that simultaneously combine PET and MRI in one system provide synergistic information in the evaluation of patients with prostate cancer and biochemical recurrence. A key benefit of combining MRI and PET is in the assessment of changes in the soft tissues, particularly in the prostate bed [133]. Image quality and anatomic localization of lesions in the prostate bed by PET/MRI are expected to be superior to PET/CT [134], which is especially the case for often very small locally recurrent tumors. Kranzbühler et al. found that ^68^Ga-PSMA-11 PET/MRI imaging demonstrated excellent detection of biochemical recurrence, including local, lymphatic, and metastatic bone disease. The overall detection rate was 78.6% in a patient population with a median PSA of 0.99 ng/mL [133,135]. Additionally, Afshar-Oromieh et al. [136] directly compared the efficacy of PSMA PET/MRI to PET/CT. The study noted that the different diagnostic sequences and higher image resolution of MRI allowed for easier identification of PSMA-positive lesions. The study noted a limitation of reduced signal at the level of the kidney and bladder [136]. A significant challenge with PET/MRI technology is the longer scan time compared with PET/CT, in part due to various organ-specific MRI sequences that can be added to the imaging protocols. The longer scanning time may not be well tolerated by patients. Conversely, PET/MRI offers lower radiation exposure than PET/CT, where the CT portion is omitted [137].

While an increasing number of studies are currently evaluating the efficacy of PSMA PET/MRI for the localization of biochemical recurrence, a detailed review of these publications is beyond the scope of this report. However, the superior anatomical detail provided by MRI raises PSMA PET/MRI as a valuable tool for detecting biochemical recurrence and guiding treatment decisions in patients with prostate cancer.

### 3.2. Future Directions and AI

Artificial intelligence (AI) and radiomics are increasingly implemented in research applied to biochemical recurrences of prostate cancer. Recently, AI-based algorithms have presented significant potential in enhancing the interpretation of PSMA imaging. AI may aid in the extraction and analysis of subtle imaging features, especially in detecting subclinical recurrences that might otherwise be missed by traditional methods. AI models may help distinguish between malignant and benign tumors by integrating radiologic and tissue pathology comparisons, potentially reducing false-positive results. However, the precision of AI-based algorithms in detecting biochemical recurrence has yet to be established. One significant problem with all AI-based models is the choice of “ground truth” algorithms used to train the AI model. Margail et al. proposed a denoising AI algorithm for ^68^Ga-PSMA-11 PET/CT in the localization of biochemical recurrence [138]. Li et al. proposed segmentation based on a deep learning convolutional neural network initially trained to identify non-suspicious structures and analyzed 193 ^18^F-DCFPyL PET/CT scans from patients with biochemically recurrent prostate cancer [139]. The authors reported that their framework outperformed both single-modal and multi-modal neural network-based approaches, demonstrating good accuracy in distinguishing suspicious foci from those unlikely to represent cancer [139]. Marturano et al. [140] studied the feasibility of radiomic analysis of ^18^F-fluoromethylcholine PET/CT in 74 patients for the evaluation of biochemical recurrences. The results of the study suggested that, compared to a clinical model based on PSA, Gleason score, and clinical stage, the prediction performance for biochemical recurrence improved when clinical data were complemented with radiomic features, particularly when a combination of prostate gland segmentation and SUV thresholding methods was applied. While the above-mentioned studies offer intriguing insights into the utility of AI models for the evaluation of biochemical recurrences in prostate cancer, their precision and reliability remain to be validated. Nonetheless, AI and radiomics approaches are uncovering previously underappreciated features of potential clinical value.

## 4. Conclusions

Diagnostic imaging techniques in prostate cancer are rapidly evolving, yet the localization of biochemical recurrence remains a challenge (Table 1 and Table 2). Initially, radiotracers such as ^11^C-choline and ^18^F-fluciclovine significantly improved the detection of recurrent disease compared with standard imaging. The longer half-life of ^18^F next enabled its commercial distribution, making ^18^F-fluciclovine widely available across the U.S. The FDA’s approval of PSMA PET imaging agents has resulted in significant advancement in the field, as PSMA radioligands demonstrate superior sensitivity and specificity to earlier radiotracers like ^11^C-choline and ^18^F-fluciciclovine. The first PSMA-targeted radioligand (^68^Ga-PSMA-11) received FDA approval in December 2020, followed by ^18^F-DCFPyL (Piflufolastat^®^ F-18 (PYLARIFY^®^) in May 2021 and Flotufolastat F-18 (POSLUMA^®^) in May 2023. These agents offer high sensitivity and specificity, often leading to cancer upstaging and significantly influencing treatment selection for patients with biochemical recurrence. Furthermore, PSMA PET/CT radioligands now play a pivotal role in identifying candidates for the PSMA-targeted radioligand therapy PLUVICTO^®^, which received FDA approval for mCRPC after androgen receptor pathway inhibitor and taxane chemotherapy in March 2022 [45], and more recently in March 2025 to include mCRPC patients who have progressed on ARPI therapy and can delay taxane-based chemotherapy [130]. Furthermore, the clinical investigation of complementary non-PSMA-based PET radiotracers, such as bombesin-based radiotracers, is underway. One compound, ^68^Ga-bombesin, demonstrates promising efficacy for the detection of local recurrence with little or no PSMA expression. The combination of sensitive and specific novel PSMA ligands, next-generation whole-body PET scanners (e.g., Biograph Vision Quadra), PET/MRI applications, and evolving AI and radiomics holds strong promise for enabling more personalized and effective treatment strategies.

## Figures and Tables

**Table 1:** PET tracers in prostate cancer, mechanism of action, imaging target, and tracer-specific advantages and disadvantages.

Radiotracer	Event	Target	Advantage	Disadvantage
^18^F-FDG	Glycolysis	glut1, glut3, hexokinase	Prognostic, aggressive disease	High urinary elimination, non-specific to prostate cancer
^18^F-Choline; ^11^C-Choline	Membrane synthesis	Choline kinase	Rapid tissue uptake	Prone to confounding uptake due to inflammation or prostatic hypertrophy
^18^F-NaF	Bone formation	Hydroxyapatite	Skeletal disease localization	Limited evaluation of soft-tissue lesions
^18^F-Fluciclovine	Amino acid transport	l-amino acid transporter	Fast tumor cell uptake and minimal, early urinary excretion	Non-specific uptake related to benign prostatic hyperplasia, infection, and inflammation
^68^Ga-PSMA-11	Transmembrane protein	PSMA	TNM staging, post-treatment evaluation	High urinary bladder activity, non-specific uptake in other tumors
^64^Cu-PSMA (DOTAGA and NODAGA)	Transmembrane protein	PSMA	High theragnostic potential, excellent image quality, radioconjugate stability	Low positron emission/not FDA approved yet
^64^Cu-PSMA-I&T	Transmembrane protein	PSMA	Uses the same conjugate for imaging and therapy	Not FDA approved yet
^18^F-DCFBC	Transmembrane protein	PSMA	Adequate tumor-to-muscle ratios	Persistent blood pool activity
^18^F-DCFPyL	Transmembrane protein	PSMA	Rapid plasma clearance, low accumulation in the liver and muscle tissues	High physiological uptake in the salivary glands, kidneys, and urinary bladder
^18^F-PSMA-1007	Transmembrane protein	PSMA	Predominant hepatobiliary excretory route	False-positive bone lesions
^18^F-rhPSMA-7.3	Transmembrane protein	PSMA	Reduced urinary excretion than ^18^F-DCFPyL and ^68^Ga-PSMA-11	False-positive bone lesions
^68^Ga-RM2	G protein-coupled receptor	Gastrin-releasing peptide receptor (GRPR)	High accuracy in identifying primary and locally recurrent disease	Slow major-vessel blood pool clearance complicating lymph node identification
^177^Lu-PSMA-617, ^225^Ac-PSMA-617	Transmembrane protein	PSMA	Therapy	Efficacy of treatment dependent on PSMA uptake

**Table 2:** Scaled stratification of various PET tracers for the management of biochemical recurrence of prostate cancer based on sensitivity and specificity. The score is indicated using (+) signs: + minimal value; ++ little value; +++ moderate value; ++++ good value; +++++ optimal value.

Radiotracer	Sensitivity	Specificity	Comments
^18^F-FDG	+	+	Limited sensitivity; highly nonspecific, given uptake in inflammation.
^18^F/^11^C-Choline	+++	++++	Good sensitivity in patients with PSA values > 2.0; specificity improved from ^18^F-FDG and ^18^F-NaF. Limited specificity due to mild uptake in inflammation and infection and uptake in non-prostate malignancies and other benign processes.
^18^F-NaF	++	+	Highly sensitive for the detection of bone metastases; however, has no role in the detection of soft-tissue lesions; highly nonspecific, given uptake in benign sclerotic lesions.
^18^F-Fluciclovine (FACBC)	++++	+++	Increased sensitivity in comparison to ^18^F/^11^C-Choline; limited specificity due to mild uptake in inflammation and infection and uptake in non-prostate malignancies and other benign processes.
^68^Ga/^18^F/^64^Cu-PSMA-11 ^18^F-DCFPyL ^18^F-PSMA-1007 ^18^F-rhPSMA-7.3	+++++	+++++	Increased sensitivity in comparison to ^18^F-Fluciclovine. False negatives in PSMA non-expressing prostate cancer (approximately 10% of prostate cancers); specificity slightly limited in patients with benign osseous lesions (particularly ^18^F-rhPSMA-7.3 and ^18^F-PSMA-1007) and uptake in non-prostate malignancies and other benign processes.
^68^Ga-DOTA-Bombesin	+++	++++	Data remain limited; however, promising alternative in cases of PSMA-negative disease.
